# Incidence and Mortality of Various Cancers in Iran and Compare to Other Countries: A Review Article

**Published:** 2018-03

**Authors:** Bagher FARHOOD, Ghazale GERAILY, Ahad ALIZADEH

**Affiliations:** 1. Dept. of Medical Physics and Radiology, Faculty of Paramedicine, Kashan University of Medical Sciences, Kashan, Iran; 2. Dept. of Medical Physics and Biomedical Engineering, School of Medicine, Tehran University of Medical Sciences, Tehran, Iran; 3. Dept. of Epidemiology and Reproductive Health, Reproductive Epidemiology Research Center, Royan Institute for Reproductive Biomedicine, ACECR, Tehran, Iran

**Keywords:** Cancer, Incidence, Mortality, Risk factor, Iran

## Abstract

**Background:**

Iran in recent years had the rapid development of industrialization and modernity, and changes in the people’s lifestyles and environment, these changes may affect epidemiological patterns of various types of cancers. In this review, incidence and mortality of various cancers (skin, gastric, esophageal, breast, and prostate) in Iran have been reported.

**Methods:**

The related data about Iran and other countries were collected from databases such as Google Scholar, Scopus, PubMed, EMBASE, and Web of Science. All included studies were published before Jun 2017.

**Results:**

There is an increment trend of incidence and mortality rate for most cancers in Iran.

**Conclusion:**

The plan for control and prevention of cancers must be a high priority for health policy in Iran as well as it is suggested that earlier screening is need for high-risk population.

## Introduction

The uncontrolled growth of cells in the body can cause variety of diseases that called cancer. It is affected by various genetic and environmental factors (1). Cancer is the main cause of death in developed countries and the second main cause of death in developing countries (2). Based on the estimation of International Agency for Research on Cancer (IARC), about 14.1 million cancer cases and 8.2 million cancer deaths have been occurred (3).

Iran is a developing country located in the Middle East, which is a region of Asia, Africa, and Europe. This country is located in a particular geo-strategic status by connecting the western and eastern parts of the world (4). Iran in recent years had the rapid development of industrialization and modernity, and changes in the people’s lifestyles and environment, these alterations may affect epidemiological patterns of various types of malignancies (5–8). In Iran, cancer is the second largest group of chronic non-communicable diseases and the third most common cause of death after heart disease, accidents and other natural phenomena (9). The age-standardized incidence rates (ASIR) of cancers were 110 and 98 per 100000 among males and females, respectively. The estimation of mortality rates for cancers was 65 and 41.1 per 100000 for males and females. The male to female standard ratio was 1.12. The most common malignancy among men and women were stomach cancer and breast cancer, respectively (10).

The incidence of cancer in various countries is different. Five common cancers (except skin cancer) in males in the world were consist of lung, prostate, colorectal, stomach, and liver, but in females included breast, lung, colorectal, cervix uteri, and stomach (3, 11). As well, “five common cancers (except skin cancer) in Iranian males were stomach, prostate, bladder, colorectal, and esophagus, and in females were breast, colorectal, stomach, esophagus, thyroid” (4).

## Methods

This paper conducted the data collected from databases such as Google Scholar, Scopus, PubMed, Embase, and Web of Science. All included studies were published before Jun 2017. The data were obtained from research and review articles on the incidence and mortality of various cancers in Iran and other countries, summarized to form a comprehensive review article. The following search terms in the titles and abstracts were used to search the databases: Cancer, Malignancy, Tumor, Incidence, Prevalence, Mortality, Skin, Gastric, Stomach, Esophageal, Breast, and Prostate, Iran.

## Results and Discussion

### Incidence and mortality of skin cancer

Skin cancer is the most frequent types of cancers worldwide and it has an increasing trend (12). In the Middle East, this is the most frequent cancer (13). The annual incidence of non-melanoma type is 2 to 3 million cases, and at least 132000 melanoma cases happen in the world (14). More than 50% of incident cases of the cancers and 33% of death happen in the low- and middle-income countries (12). Furthermore, the incidence of melanoma is rising with the age and it is more prevalent in the eighth decade of life. However, the occurrence in young people (under 30 yr old) is not rare, which makes it prevalent cancer among the young people (15). Skin cancer is also the most prevalent cancer in Iran (16) with a male-to-female ratio of 1.6 (17). This cancer is the first most frequent cancer for males with ASR of 18.93 and the second most frequent cancer for females with ASR of 13.09 (18). Overall, 7000 new cases are detected every year in Iran. In addition, the most common type of skin cancers is NMSC, composing 95% of skin malignancies (19). In 2004, the incidence rate of this cancer is 10.13 per 100000 people (20). Around 15% of all malignancies were skin cancer in Iran (21). In another study, the lowest and highest ASR were found in the 0–4 and 80–84 age groups, respectively. In addition, the most common skin cancer in Iran is BCC, which its incidence rates in males and females decreased from 69% to 59.1% and 75.9% to 65.9%, respectively (22). In another study, there had a decreasing trend for BCC, but SCC and melanoma had a rising trend. The mortality rate has declined from 0.546 per 100000 in 2006 to 0.522 per 100000 in 2010. Moreover, female to male ratio of mortality rate was more than one during the years studied (2006–2010) and mortality rate is more among the women than men (18). The increment in population, the proportion of the elderly population and life expectancy, and lifestyle change and socioeconomic status leads to Iran is considered as the country at high risk of skin malignancies (12).

In a study on epidemiology of skin cancer incidence, this cancer had highest ASR in provinces of Fars, Khuzestan, Bushehr, and Hormozgan. Moreover, provinces of southern in Iran had high incidence and its incidence was higher in men than females. It may be because of increased exposure to sunlight, increment of eldery population, exposure to risk factors, benefit from better diagnostic facilities, refer to detect cancer from surrounding provinces, etc. (16). In another study, the incidence of NMSC in the Isfahan Province was much less compared to western countries; as its incidence was 10.67 per 100000 individuals (23). In another study, Sanandaj with 17.55 per 100000 and SarvAbad with 3.73 per 100000 had the highest and the lowest incidence rates in Kurdistan Province (24). In another study in Tehran, skin cancer incidence had a rising trend and its incidence rate has raised from 8.99 in females and 11.52 in males in 2003 to 18.03 in females and 28.16 in males in 2008, respectively. In addition, incidence of this cancer was raised with aging in both genders; however, the incidence rate was higher in males than females. Therefore, increment of cancer incidence in Tehran Province could be because of environmental risk factors, population aging, and changes in lifestyle (25).

High incidence of skin malignancy and its relationship to sunlight exposure, educating people on the true sun protection, empowering and educating physicians and the people about early detection of this cancer seems to be significant in preventing its prevalence. However, if this cancer is timely diagnosed, it could be successfully treated. Furthermore, removing the predisposing factors could decrease the prevalence of these cancers.

### Incidence and mortality of gastric cancer

Gastric cancer is the fifth most common diagnostic cancer and the third most common mortality factor among malignancies throughout the world (26). Overall, the mortality and incidence of gastric cancer have decreased dramatically in the past 70 yr (5). In Iran, it is the most frequent cancer in males and the second common cancer in females (after breast cancer) (27). Mortality of this cancer in both sexes, gastric cancer is the most common cancer cause of death in Iran (27). There is an increment in the incidence of this cancer in Iran, particularly in the Northern provinces like Mazandaran (28). Generally, patient age is an important prognostic factor of gastric cancer, the more patient age at the diagnosis time, the poorer prognosis of the patients. Changes occurred in the mean age of the population of Iran can be affected the prevalence of this cancer as well as its prognosis. Another main problem related to gastric cancer is that most patients in late disease stages are diagnosed, therefore, it is effectively difficult to treat them and this delay in the cancer diagnosis significantly reduces survival time of the patients (27). Recently, a great prevalence of *Helicobacter pylori* infection, smoking, gastroesophageal reflux disease, and high dietary intake of salt are the significant environmental factors of this cancer in Iran (29).

In Iran, this cancer is of great prevalence in the northwest and north (particularly in Azeri-speaking provinces), low prevalence in the southern areas, and a moderate prevalence in the center and western areas. Kerman and Ardabil provinces had the lowest (1.5 men and 10.2 women in 100000) and the highest (25.4 men and 49.1 women in 100000) incidence, respectively (30). The great incidence of gastrointestinal cancers in the northwest and north regions of Iran can be justified with respect to a supposed belt of upper gastrointestinal tract cancers (including esophagus and stomach) which drives from the far East (China, Korea, and Japan) and passes the central Asian countries (Turkmenistan and Uzbekistan) and the Near East (Iran and Eastern Anatolia region of Turkey and Caucasus). Kurdistan provinces and West Azerbaijan of Iran are placed on the belt (30). The provinces of Semnan, East Azerbaijan, and Golestan, as well as Tehran metropolitan region, also have high rates of stomach cancer in both women and men (31). In another study, a lower incidence rate of gastric cancer with age-standardized rate (ASR) of 5.1 and 10.2 in women and men for Kerman province was reported (32, 33). In addition, the greater incidence rate of gastric cancer with an incidence of 8.6 and 26.4 in females and males respectively was reported in Ardabil that it might be because of higher rates rather gastric cardia than non-cardia cancer (29). Furthermore, age-standardized mortality rates for stomach cancer in population of Ardabil were 32.2 and 16.3 (34). The findings of a study in Ardabil Province demonstrated that H. pylori infection is remarkably lower in the central region of Iran in comparison with those of Ardabil Province (the north-west region of Iran) (35).

The incidence of stomach cancer is rising in Iran; epidemiological studies are essential steps into the etiology and early detection of this cancer (5).

### Incidence and mortality of esophageal cancer

Esophageal cancer is the eighth most frequent malignancy as well as the sixth leading cause of death in the worldwide (3). Incidence of EC varies widely throughout the world; with ASR per 100000 over 20 in Eastern Asia and in Southern Africa to as low as 1.4 as in Western Africa (36). In another report, EC is the third most common gastrointestinal cancer and demonstrates high incidence in parts of Iran (37). With regards to westernization and lifestyle changes in Iran and other developing countries, the incidence of this cancer can be similar to the developed countries. In addition, the improvement of public health, as well as epidemic of gastrointestinal reflex diseases and obesity may be the reason for high incidence of esophageal cancer in Iran (38). In a report by Iran Cancer Institute, 27% of gastrointestinal malignancies and 9% of all malignancies were esophageal carcinoma, as a male to female ratio was 1.7:1 (31). Iran is a country with intermediate risk; as ASR is around 7 for males and females. However, some regions of Iran are located in the Asian countries with high incidence of EC, as ASR is as high as 100 (36). In another study, EC is the second and third most frequent cancers in Iranian men and women, respectively (39).

The counties in provinces of Ardebil, Kordestan, and Mazandaran have higher risk than other counties (37). In addition, Golestan Province in northeastern of Iran is one of the higher risk regions of the world, followed by Provinces of Mazandaran and Khorasan (31). The incidence rates of EC during 2005–2006 were 6.25 and 5.83 in women and men, respectively (10). In a study, the ASIR for esophageal cancer in Northern, Northeast Western, and Northwest provinces of Iran were higher than other provinces. They found the highest ASIR in both sexes in the 80–84 year age group with 114.5 in women and 147.5 in men (39). In a study in Golestan Province, a low in-take of fresh vegetables and fruits, opium consumption, and low socioeconomic status are associated with a higher risk of EC. Furthermore, studies have focused towards the probable role of drinking very hot tea (40, 41). In addition, the reason of high incidence in north part is nitrate including soil and special nourishing in those areas (37). Recently, there are several reports on estimating the EC incidence rate in some province of Iran (42–45).

Public education, eradication of opium addiction, and nutritional support may decline the mortality and morbidity that results from EC (46).

### Incidence and mortality of breast cancer

Breast cancer (BC) is the most common cancer and the leading cause of death from cancers among women (3). There are different reports in the incidence rates of BC throughout the world. In a study highest incidence rates are in North America with ASR 123.6 per 100000 and Western Europe with ASR: 84.6 per 100000, intermediate incidence rates in the South American and Mediterranean countries with ASR: 46 per 100000, and lowest incidence rates in Southeast with ASR: 25.5 per 100000 and South Central Asia with ASR: 21.8 per 100000 (47). This cancer incidence in developed countries is higher than developing countries, because of higher prevalence of the cancer risk factors in developed countries, such as low parity, older age at first pregnancy, sedentary occupation, high-calorie intake, and use of hormonal replacement therapy. However, BC survival is lower in less wealthy countries and in females with educational level or low income (48). Global incidence trend of this cancer is rising particularly in countries with a low incidence rate and Iran country is not an exception (49). In Iran, BC ranks first among malignancies diagnosed in females, with comprising 24.4% of all cancers with ASR of 23.1 per 100000 (48, 49), and is the fifth most common causes of death related to cancers (48). According to the new statistics in Iran, 6160 breast malignancies are diagnosed in the country each year and 1063 cases cause death (50). Iran as developing country faces with an increment in BC (21, 48, 49). According to a study in Iran, oral contraceptives usage, low parity and employment, and family history of breast cancer can be affected by increased risk of BC (51). In addition, In Iran, there is not a national screening program early diagnosis and to control of BC, because early detections of BC is the significant factor for decreasing its burden (48, 49). Furthermore, the females’ awareness of BC warning signs and impressive screening are very insufficient (48). The incidence rate of BC is different in different areas of Iran by the lifestyles, age composition of populations, and behaviors of individuals (21). In the first report related to incidence and age distribution of BC in Iran, ASR was calculated 17.1 based on population-based data from five provinces (Mazandaran, Gilan, Golestan, Kerman, and Ardabil) (52). According to a report in 2005, the lowest and the highest rates have been reported in Chaharmahal and Bakhtiari and Tehran provinces, respectively (30). BC is also the most frequent malignancies in women of Tehran and its mortality rate increased (49). According to a report in 2003 related to the annual incidence rate of BC, the provinces of Yazd, Khorasan, Fars, Qom, and Khuzestan are next to Tehran province, respectively (30). By increasing females’ social role in urban regions, social effects of exposure to risk factors of the cancer can be efficacious in development of the cancer, as it is more frequent in large cities such as Tehran and Yazd in compared to provinces away from Tehran like Hormozgan and Kurdistan with different lifestyle and lower population densities. In a study, there is a positive relationship between BC with socioeconomic development (30). Another study in Kerman Province showed that the BC incidence has increasing trends in both genders which this may be due to changes in lifestyle, increase of life expectancy, and increasing exposure to risk factors of cancer (30).

Early detection of BC plays the important role in improving the patients’ prognosis and reducing mortality among women (49). There is an increasing trend for BC mortality in Iran. Thus, programs of health education to inform females regarding the risk factors and signs, and national screening to help early diagnosis are required for the women community in Iran (49).

### Incidence and mortality of prostate cancer

One of the main cancers is prostate cancer (PC); so that it is the second most frequent cancer in males in the world (53) and the first most frequent cancer in European and American males (54), while 1.1 million males suffered from this cancer in the world, around 70% in developing (3). Nearly 42% of PC cases happen in males over 50 yr (55) and most cases often are observed after 60 yr (56, 57). The highest death rates related to PC were observed in African-American race which the global incidence was of 25.3 per 100000 and ASR was 137 per 1000000; as were 60 times higher than the incidence reported in Shanghai, China with the lowest rate in the world i.e. 2 per100000 people (1). Geographical distribution of this cancer is different; as in the Asian countries (including Iran) is less than in Western countries (58). Their reason might be because of the availability of screening tests applied for diagnosis like prostate specific antigen (PSA) test, and other factors such as nutrition, lifestyle, genetics, environmental factors, race, smoking, physical activity, and cancer registry systems (57). In Iran, PC has increased in the past few decades, as it was at rank of thirteenth in 1986 and reached the rank of fourth in 2005; as a portion of this rising is related to the increment in early detection of PC cancer (PSA test) and a portion to the real increment in its prevalence due to risk factors related to lifestyle (59). The incidence of this cancer among population of Iran was severely higher than other countries of Asia (60). According to the first report related to incidence of PC in Tehran, this cancer has an ASR of 15.6 and it is the second most frequent cancer among males in Tehran (61). Moreover, data obtained from cancer registry reported during 1996–2000 in five provinces (Mazandaran, Gilan, Golestan, Tehran, and Ardabil), the ASR was calculated 5.1 (30), while the incidence reached to 12.5 in 2013, so it represents a rising trend in the incidence of this cancer. Over 65 yr, the incidence of this cancer rises significantly; this can be because of the effects of aging as well as the accumulation of risk factors over time. Because the high age-specific incidence rates in this study were seen in age over 60, older age is considered as the powerful risk factor in great incidence areas (3). In a recent study in 2016, PC is the second most frequent cancer in males in the world as well as the third most frequent cancer in males and the sixth most frequent cancer in Iran (58). The geographical distribution of this cancer showed that Tehran had the highest prevalence, followed by in provinces of Isfahan, Mazandaran, and Fars, as well as the lowest prevalence in Ilam Province (30). In several studies in various province of Iran, ASIR in Ardabil, East Azerbaijan, and Semnan provinces were 3.5, 7.33 and 10.11 per 100000 people, respectively (58, 62, 63) and in the Fars Province was indicated that PC is not one of the ten most frequent cancers. Age and nutrition can be effective factor in developing this cancer. Therefore, because of changes in older population and nutrition, it can be expected to modify over time the incidence of PC (58).

## Conclusion

There is an increment trend of incidence and mortality rate for most cancers in Iran. Therefore, the plan for the control and prevention of cancers must be a high priority for health policy as well as it is suggested that earlier screening is need for high risk population.

## Ethical considerations

Ethical issues (Including plagiarism, informed consent, misconduct, data fabrication and/or falsification, double publication and/or submission, redundancy, etc.) have been completely observed by the authors.
